# Ketamine Combined With Psychotherapy as a Treatment for Resistant Depression in a Public European Hospital

**DOI:** 10.1002/brb3.71164

**Published:** 2026-01-13

**Authors:** Filipa Alves da Silva, Rita Avelar, Bernardo Peixoto, Leonor Bacelar‐Nicolau, Francisco Santos, João Costa Ribeiro, Maria João Heitor

**Affiliations:** ^1^ Department of Psychiatry and Mental Health, Hospital Beatriz Ângelo Unidade Local de Saúde (ULS) Loures‐Odivelas, E.P.E. Loures Portugal; ^2^ Instituto de Medicina Preventiva e Saúde Pública, Faculdade de Medicina Universidade de Lisboa Lisboa Portugal; ^3^ Environmental Health Behaviour Lab, Instituto de Saúde Ambiental and Laboratório Associado TERRA, Faculdade de Medicina Universidade de Lisboa Lisboa Portugal; ^4^ Laboratório de Bioestatística, Faculdade de Medicina Universidade Católica Portuguesa Lisboa Portugal; ^5^ Centro de Filosofia das Ciências Universidade de Lisboa Lisboa Portugal; ^6^ Centro de Investigação Interdisciplinar em Saúde–CIIS, Faculdade de Medicina Universidade Católica Portuguesa Lisboa Portugal; ^7^ Faculdade de Medicina Universidade Católica Portuguesa Rio de Mouro Portugal; ^8^ Portuguese Society of Psychiatry and Mental Health–SPPSM Lisboa Portugal

**Keywords:** ketamine, off‐label ketamine, psychedelic‐assisted therapy, psychotherapy, resistant depression, response rates, treatment

## Abstract

**Purpose:**

Depression affects around 280 million people worldwide, and about 30% of patients have treatment‐resistant depression. Ketamine has significant scientific evidence supporting its use as an antidepressant, making it a promising approach for treatment‐resistant cases. Combining ketamine with psychotherapy may enhance therapeutic response and support longer‐lasting cognitive and behavioral change. This pilot proof‐of‐concept study aims to evaluate the effect of treatment with ketamine infusion combined with psychological intervention in a sample of nine patients with treatment‐resistant depression at a general hospital within the Portuguese National Health Service.

**Methods:**

Clinical outcomes were measured through the clinical interview and the patient health questionnaire (PHQ‐9) to assess complete or partial improvement.

**Results:**

Following eight weeks of treatment, all showed a reduction in their PHQ‐9 scores, with the median score transitioning from a baseline categorization of “severe” depression to a “moderate” level. It was found that 44.4% (4/9) of participants showed a response to treatment (≥ 50% reduction in the PHQ‐9 score). Among the patients with suicidal ideation, slightly over half showed remission of these thoughts at the end of treatment. Among the participants subsequently monitored as outpatients, only 29% (2/7) experienced a deterioration in mood within three months post‐treatment, requiring an adjustment of antidepressant therapy.

**Conclusion:**

In our study, an improvement in depressive symptoms was observed, despite their severity, in a sample submitted to multiple previous pharmacological strategies.This retrospective study evaluated ketamine infusions combined with psychotherapy in nine patients with treatment‐resistant depression at a general hospital. After eight weeks, all participants improved, with PHQ‐9 scores shifting from severe to moderate. Overall, 44% responded to treatment, and among those with suicidal ideation, more than half showed remission.

## Graphical abstract

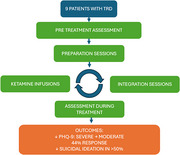

## 1 Introduction

Depression affects around 280 million people worldwide, developing in all age groups and it is one of the main causes of disability (World Health Organization 2021). Around 30% of patients with this condition have treatment‐resistant depression (TRD) (Rush et al. 2006, McLachlan 2018). The definition can be summarized as a lack of response to successive antidepressant therapies.

There is a clear need to develop effective treatments for TRD. Psychiatrists currently have different strategies that have accumulated evidence in the treatment of resistant cases, including electroconvulsive therapy (ECT), trans cranial magnetic stimulation (TMS), and the use of ketamine and esketamine (Berman and Ambrose 2022, Anand et al. 2023). ECT remains the most effective intervention for TRD, but its use is limited by stigma, limited accessibility, and concerns about cognitive side effects. (Mathew et al. 2019) Recent meta‐analytic findings suggest that intravenous ketamine and repetitive trans cranial magnetic stimulation (rTMS) may offer comparable efficacy and greater acceptability, although the overall certainty of the evidence remains low, highlighting the need for further high‐quality trials (Terao et al. 2025).

Ketamine has shown evidence of efficacy and safety in the various studies published over the last 20 years, making it a promising approach in the treatment of resistant depression (Sanacora et al. 2017, Andrade 2017). It can be used individually or integrated into a psychedelic‐assisted therapy (Greenway et al. 2020, Garel et al. 2023). Pharmacological and psychological interventions are combined to use the psychoactive effects of ketamine. These properties include its antidepressant and anxiolytic effects, acute subjective effects, and enhancement of psych plasticity, thereby fostering the ability for psychological and behavioral adaptations (Muscat et al. 2021, Vargas et al. 2021, Olson 2018).

Despite robust support for ketamine in treatment‐resistant depression, most studies come from private or research settings, leaving a gap in understanding how ketamine‐based treatments perform in public European healthcare services, where resources and patient profiles differ.

### 1.1 Clinical Pharmacology of Ketamine

Ketamine was first studied as an anesthetic drug, producing a dissociative state associated with profound analgesia, with a limited duration of action and a low side‐effect profile (Li and Vlisides 2016). Its antidepressant action is primarily linked to a non‐competitive antagonist of N‐methyl‐D‐aspartate (NMDA) receptors, with selectivity for inhibitory GABAergic interneurons and subsequent glutamatergic activation. In addition, the involvement of other mechanisms of action and other monoaminergic pathways are being considered (Matveychuk et al. 2020).

Adverse effects are usually transient and benign, the most common being: dissociation, headache, dizziness, elevated blood pressure, and blurred vision (Short et al. 2018). Although chronic recreational use has been associated with urological toxicity, current evidence from clinical studies suggests that the risk of urinary or renal complications in psychiatric treatment settings is low (Kerr‐Gaffney et al. 2025).

Psychiatric indications for the use of ketamine include depression, generalized anxiety disorder, social phobia, post‐traumatic stress disorder, alcohol, cocaine, or opioid use disorder, obsessive‐compulsive disorder and eating disorders, with different levels of evidence in the literature (Walsh et al. 2022, Grabski et al. 2022).

### 1.2 Ketamine as an Antidepressant

The first randomized controlled study was published in 2000 (Berman et al. 2000), and there is currently meta‐analytical evidence (Marcantoni et al. 2020, Kryst et al. 2020).

A single infusion of ketamine produces a rapid and robust response in at least 50% of patients. The effect of a single dose is transient, dissipating between 10 and 14 days, although it can be prolonged with multiple infusions (Kishimoto et al. 2016, Coyle and Laws 2015).

Meta‐analytical findings from clinical trials, alongside recent real‐world data, indicate response rates to ketamine treatment of approximately 50% and symptom remission rates of about 28%, persisting up to 31 days post‐last infusion in depression patients (Marcantoni et al. 2020, McInnes et al. 2022). When compared to the efficacy of antidepressants in resistant depression, there are higher response and remission rates with the use of ketamine. A study published in 2011 reported 27% response and 17% remission rates with the use of an antidepressant at six months after two ineffective treatments (Heerlein et al. 2022). A 2006 study also reported 14% remission rates with the use of antidepressants as the third line of treatment, while 32.9% and 30.6% were reported when used as the first or second line, respectively (Rush et al. 2006).

The benefits of using ketamine as an antidepressant have mostly been studied in resistant depression. However, its use is also effective in bipolar depression despite the scarcity of studies (Bahji et al. 2021, Wilkowska et al. 2020).

### 1.3 Ketamine as a Psychedelic

The acute psychological effects of ketamine include a partial loss of contact with external reality, where various experiences are described, such as emotionally intense visions, a feeling of leaving the body, a deep sense of happiness and peace, a feeling of loss of identity, and encounters with unearthly or sacred beings (Kolp et al. 2014). Although ketamine has mostly been used without the psychotherapeutic component, and in this context, these effects are considered undesirable, it has also been used as an adjunct to psychotherapy and as a form of psychedelic‐assisted psychotherapy since the 1970s (Dore et al. 2019).

### 1.4 Ketamine‐Assisted Therapy

The combination of psychotherapy with psychedelics can lead to emotional, cognitive, and behavioral changes (Schenberg 2018). Several psychedelic compounds, such as LSD and psilocybin, appear to increase neuroplasticity, both during the substance exposure sessions and in the days afterward (Ly et al. 2018, Cavarra et al. 2022, de Vos et al. 2021). There seems to be an opportunity for the use of psychotherapy to facilitate the modification of rigid behaviors, thought patterns, and emotional reactions (Cavarra et al. 2022). Ketamine has a psychoplastogenic effect that stimulates synaptogenesis and increases neuroplasticity (Muscat et al. 2021, Vargas et al. 2021, Olson 2018). The combination of ketamine with psychotherapy may be associated with enhanced therapeutic effects, potentially supporting more lasting cognitive restructuring and behavioral change (Hasler et al. 2020).

Psychedelic‐assisted therapy involves the use of a psychedelic compound within a limited set of therapy sessions designed to guide and interpret the psychedelic experience (Nielson 2018). It should encompass four phases: clinical assessment, preparation, dosing session/experience, and integration (Greenway et al. 2020, Watts and Luoma 2020, Nutt et al. 2020).

The assessment phase serves as a screening stage to see if the individual is suitable for the treatment.

In the preparation phase, the treatment procedure is explained and a relationship of trust is established between the patient and the therapist(s) (Johnson et al. 2008). Intentions are set, allowing the guidance of the therapeutic process to meet the patient's goals and expectations (Watts and Luoma 2020, Leary et al. 2007). The person is also prepared with relaxation and focus strategies to overcome challenging experiences. The complete process is outlined to the patient, aiming to minimize anxiogenic factors and familiarize the patient with the therapeutic process and its phases (Johnson et al. 2008, Lane et al. 2015).

The experience phase is the only phase that involves ketamine and focuses only on the experience. Therapists should have a non‐directive role (Johnson et al. 2008). According to Hoffer (1965), listening to music is a key element during the ketamine infusion, as it can act as a guide, helping the experience unfold in a nonverbal way and enhancing the experience itself.

A large part of the psychotherapeutic work is done in the integration phase. Therapists and the patient work together to try to decode and interpret the experiences of the infusion phase. It helps people integrate these learning's into their day‐to‐day lives, which leads to longer‐lasting therapeutic benefits (Johnson et al. 2008; Grof 2008). This process can include exploring emotions and thoughts that have arisen in the infusion session, addressing questions or concerns, and developing strategies to maintain the gains achieved during the sessions (Grof et al. 1973; Ross et al. 2016). These experiences can help the patients generate different perspectives that allow them to integrate new meanings, values, and attitudes about the world and themselves (Krupitsky et al. 1997; Kolp et al. 2007). Various psychotherapeutic models have been used to structure the intervention. We based our intervention on the acceptance and commitment therapy model used by Yale University in Psilocybin‐Assisted Therapy for Depression (Sloshower et al. 2020, Guss et al. 2020), with growing evidence in the treatment of depression (Bai et al. 2020) and on an experience‐centered approach to integration by the MIND Foundation (2020). Although our intervention followed an ACT‐based model, other approaches—such as CBT, mindfulness‐based interventions, and motivational enhancement therapy—have also been used in combination with ketamine. However, as reviewed by Drozdz et al. (2022), the evidence remains limited and heterogeneous, and it is not currently possible to recommend one psychotherapeutic modality over another (Drozdz et al. 2022). A recent systematic review of psychedelic‐assisted psychotherapy similarly found that psychological interventions are implemented heterogeneously across studies, with no consensus on best practice for the psychotherapeutic component (Seybert et al. 2025).

In Portugal, ketamine is approved as an anesthetic, its use is considered off‐label when administered for other indications, and it is mandatory to obtain informed consent for its use in resistant depression (Ordem dos Médicos 2023). Therapies involving psychedelics pose unique challenges, particularly regarding ethical and regulatory considerations. During the psychedelic experience, the presence of alterations in the state of consciousness, increased suggestibility, and vulnerability can have consequences that are difficult to explain and anticipate. It is important to obtain informed consent before the first administration, detailing all the potential effects that may arise (Seybert et al. 2023).

### 1.5 Treatment Optimization Parameters

Ketamine can be administered through various routes, with intravenous (IV) infusion being the most common in clinical research, typically at a dose of 0.5 mg/kg over 40 min (Terao et al. 2025, Grabski et al. 2022, Seybert et al. 2023, Sakurai et al. 2020, Kroenke 2012). Although its antidepressant effects are often transient, repeated administrations have been shown to enhance and prolong therapeutic benefits (Grabski et al. 2022, Seybert et al. 2023). Treatment protocols commonly include two to three infusions per week, with weekly sessions used for maintenance in responders (Sakurai et al. 2020, Kroenke 2012, Turkoz et al. 2021). While relapse typically occurs within two to three weeks after stopping treatment in clinical trials (Kroenke 2012, Hartogsohn 2016), real‐world data suggest longer durability, with 80% of patients maintaining response at four weeks and 60% at eight weeks post‐treatment (McInnes et al. 2022).

### 1.6 Main Objective

The study aims to evaluate the effect of treatment with ketamine infusions combined with psychological intervention in a sample of patients with resistant depression in a national health service hospital. Clinical outcomes were measured through the clinical interview and the PHQ‐9 to assess complete or partial improvement.

## 2 Methods

At the Psychiatry and Mental Health Department of a general hospital from the Portuguese National Health Service (NHS), in the metropolitan Lisbon area, the following actions were undertaken: (1) Review of the literature on indications, contraindications, efficacy, safety and technical procedures associated with the use of ketamine in TRD; (2) develop the framework for the therapeutic program; (3) articulate with the Medical Day Hospital; (4) define the pathway of off‐label use with the hospital pharmacy and its therapeutics committee and the hospital ethics committee; (5) prepare the clinical protocol for hospital use; (6) create an informed consent form and information leaflet for the user; (7) provide training to the clinical team responsible for administering the treatment; and (8) select appropriate instruments for measuring clinical outcomes.

### 2.1 Clinical Protocol

The treatment included adult patients with a diagnosis of TRD defined by a lack of response to at least three antidepressants (including a selective serotonin and noradrenaline reuptake inhibitor and a tricyclic) and an augmentation strategy, with a score ≥ 10 on the PHQ‐9. Patients with psychotic symptoms, pregnant or breastfeeding women, current drug abuse and/or positive urinary toxicology, a history of adverse effects or allergy to ketamine, and unstable organic comorbidities were excluded. Patients were referred to the program via outpatient clinics, inpatient clinics, or the emergency room.

The treatment consisted of three different phases: (1) pre‐treatment assessment; (2) ketamine infusion sessions; and (3) preparation sessions and psychological integration of the experience. The protocol included one 2‐h preparation session (covering patient history, psychoeducation, and intention setting), eight acute ketamine sessions delivered over four weeks, and four maintenance sessions. Integration sessions aimed to help patients translate insights from the ketamine experience into concrete, value‐based actions through mindfulness, expressive, and reflective exercises. All therapists involved in the KAP protocol were board‐certified mental health professionals. The main therapists received specific training consisting of six hours of theoretical instruction on KAP and the manualized protocol, as well as practical training through observation and supervised practice. No formal fidelity checks were conducted. Full details of the manualized protocol are available in Supplementary Material 2 (SM‐2).

#### 2.1.1 Pre‐treatment Assessment

Two independent psychiatrists carried out the pre‐treatment assessment to determine whether the patient was eligible for treatment. Patients with severe depression, significant symptoms of psychomotor inhibition, or psychotic symptoms were considered eligible for ECT treatment instead. Informed consent was signed in this phase.

#### 2.1.2 Ketamine Infusion Sessions

The treatment was divided into two phases: acute and maintenance. The acute phase of treatment lasted four weeks, corresponding to a total of eight sessions, with two weekly administrations of ketamine. The maintenance phase corresponded to one weekly administration of ketamine, for a total of four sessions (Figure 2 in Supplementary Material 1). The total dose of ketamine was diluted in saline solution to a volume of 250 mL and administered using an infusion pump over 40 min, corresponding to a rate of 375 mL per hour. Treatment began with a low dose of ketamine (e.g. 0.25 mg/kg), which is progressively increased according to tolerability. The aimed dose of ketamine was 0.5 mg/kg IV, adjustable between 0.1 mg/kg and 1.0 mg/kg. Three blood pressure checks are carried out during the session. In addition to blood pressure monitoring, patients were clinically observed throughout each session, including heart rate, respiratory rate, peripheral oxygen saturation, level of consciousness, and brief mental‐status checks. No serious adverse events were reported during treatment. A summary of self‐reported adverse events, collected through session‐by‐session questionnaires, was provided in Supplementary Material 1.

#### 2.1.3 Preparation Sessions and Psychological Integration of the Experience

At least two preparation sessions were carried out before the first administration of ketamine. These sessions aim to accomplish the following objectives: (1) foster a therapeutic relationship and build trust; (2) identify psychological rigidity mechanisms; (3) evaluate and endorse expectations about the experience; (4) facilitate the delivery of the experience; and (5) establish intentions for therapy.

Integration sessions were held weekly. In these sessions, the aim was to integrate what had been experienced and learned in the ketamine dosage sessions.

### 2.2 Data

A total of 13 participants underwent treatment with ketamine‐assisted therapy between 05‐02‐2021 and 16‐04‐2024. More than 130 ketamine infusion sessions were carried out. After the implementation of the treatment program, a retrospective analysis was conducted on the clinical outcomes of patients treated. From this group of patients, a sample of nine participants was selected as they underwent all the assessments according to the established protocol. Four additional patients also completed the full treatment protocol but were excluded from the analysis due to missing PHQ‐9 forms. At baseline, these patients had a mean age of 53 years (range 34–68), three were female, and all had treatment‐resistant depression (see supplementary material 1). All nine participants thus completed PHQ‐9 assessments at baseline, four weeks, and eight weeks, resulting in complete data with no missing values. No imputation methods were necessary. Given the small sample size and exploratory nature of the dataset, the present report should be understood as a retrospective proof‐of‐concept pilot.

Patient characterization data collected at baseline, through clinical files, included sociodemographic (age, gender, employment status, marital status, and education) and clinical information at the time of referral (main diagnosis, presence of comorbidities, history of hospitalizations, frequency of day hospital visits and history of suicide attempts).

The PHQ‐9 was performed at baseline, after four weeks and after eight weeks of treatment.

Following the program's conclusion, the period duration until relapse was evaluated through clinical records and follow‐up appointments. Participants who required adjustments to their antidepressant therapy due to the severity of their symptoms were classified as having experienced relapse. Clinical assessments were conducted at three intervals: from zero to three months, from three to six months and from six to 12 months.

### 2.3 Evaluation Tools

PHQ‐9 is an instrument used to assess symptoms of depression and their severity through a self‐completion questionnaire with nine items referring to the presence of symptoms in the last two weeks. Absence of depression is defined if the score is between 0 and 4; mild depression between 5 and 9; moderate depression between 10 and 14; moderately severe depression between 15 and 19, and severe depression between 20 and 27 (Costi et al. 2016). A response to treatment for depressive symptoms was characterized by a reduction of ≥ 50% in the PHQ‐9 score, while remission was defined as a score of < 5 on the scale (Williams et al. 2018). We used the validated Portuguese version.

### 2.4 Statistical Analysis

Descriptive statistics were used to characterize the sample of patients (*N* = 9) univariately: absolute and relative frequencies (counts and percentages, respectively) for qualitative variables; means, standard deviations (SD), medians, and interquartile range (IQR) for quantitative variables. Given the small sample size, non‐parametric tests were used to compare the outcome PHQ‐9 at baseline and on weeks 4 and 8 of treatment: Friedman's Test was used for the quantitative score; Cochran's Q Test was applied for the binary variable recoded by severity depression classes; respective pairwise comparisons significance values were adjusted by the Bonferroni correction for multiple tests. Effect sizes for PHQ‐9 changes regarding the Friedman's Test were assessed by calculating the Kendall's W, although the small sample size limits their interpretation, and effect sizes for pairwise comparisons were calculated using rank‐biserial correlation. Additionally, 95% confidence intervals for medians were calculated for outcome PHQ‐9 scores. 95% Confidence intervals for pairwise differences in proportions were estimated using the Newcombe method for PHQ‐9 and PHQ‐9 item 9 binary recorded classes compared over time. The significance level was set at 5%. All analyses were conducted using the software IBM SPSS Statistics V.28.0.0.0 (190).

## 3 Results

### 3.1 General Characteristics

Most participants were female (6 out of 9 patients), and single (5 patients), dividing quite uniformly throughout employment status and education levels (Table 1). Concerning clinical information at baseline, nearly half the patients (4 out of 9 patients) presented a concomitant psychiatric disorder, have a hospital admission and have a history of suicide attempts. Two of the participants attended a psychiatric day hospital (two out of nine patients).

**TABLE 1:** Sociodemographic and clinical information at baseline.

—		Freq.	%^a^
Sociodemographic information		—	—
Age	Mean (SD)	42.78 (12.94)	
	Median (IQR)	44.00 [38.00; 51.00]	
Sex	Female	6	66.67%
	Male	3	33.33%
Current employment status	Employed	4	44.44%
	Unemployed	5	55.56%
Current marital status	Single	5	55.56%
	Married	2	22.22%
	Divorced	1	11.11%
	Widowed	1	11.11%
Education completed	Grade 9	3	33.33%
	Graduated highschool	2	22.22%
	University degree	4	44.44%
Clinical information at baseline			
Concomitant psychiatric disorder	Yes	4	44.44%
	No	5	55.56%
Hospital admission	Yes	4	44.44%
	No	5	55.56%
Attendance at Day Hospital	Yes	2	22.22%
	No	7	77.78%
History of suicide attempt	Yes	4	44.44%
	No	5	55.56%
Ethnicity	White/European	9	100.00%
Prior ECT/TMS	YES	0	0.00%

*Note*: ^a^Percentages should be interpreted with caution, given the small sample size.

All participants presented a lack of response to at least three antidepressants (including a selective serotonin and noradrenaline reuptake inhibitor and a tricyclic) and a potentiation strategy (i.e., the use of an adjunctive agent such as lithium or antipsychotics to enhance antidepressant efficacy), except for one participant diagnosed with bipolar disorder type II who was included in the treatment despite not having been treated with a tricyclic antidepressant.

### 3.2 Results of the PHQ‐9 Scale

**FIGURE 1:**
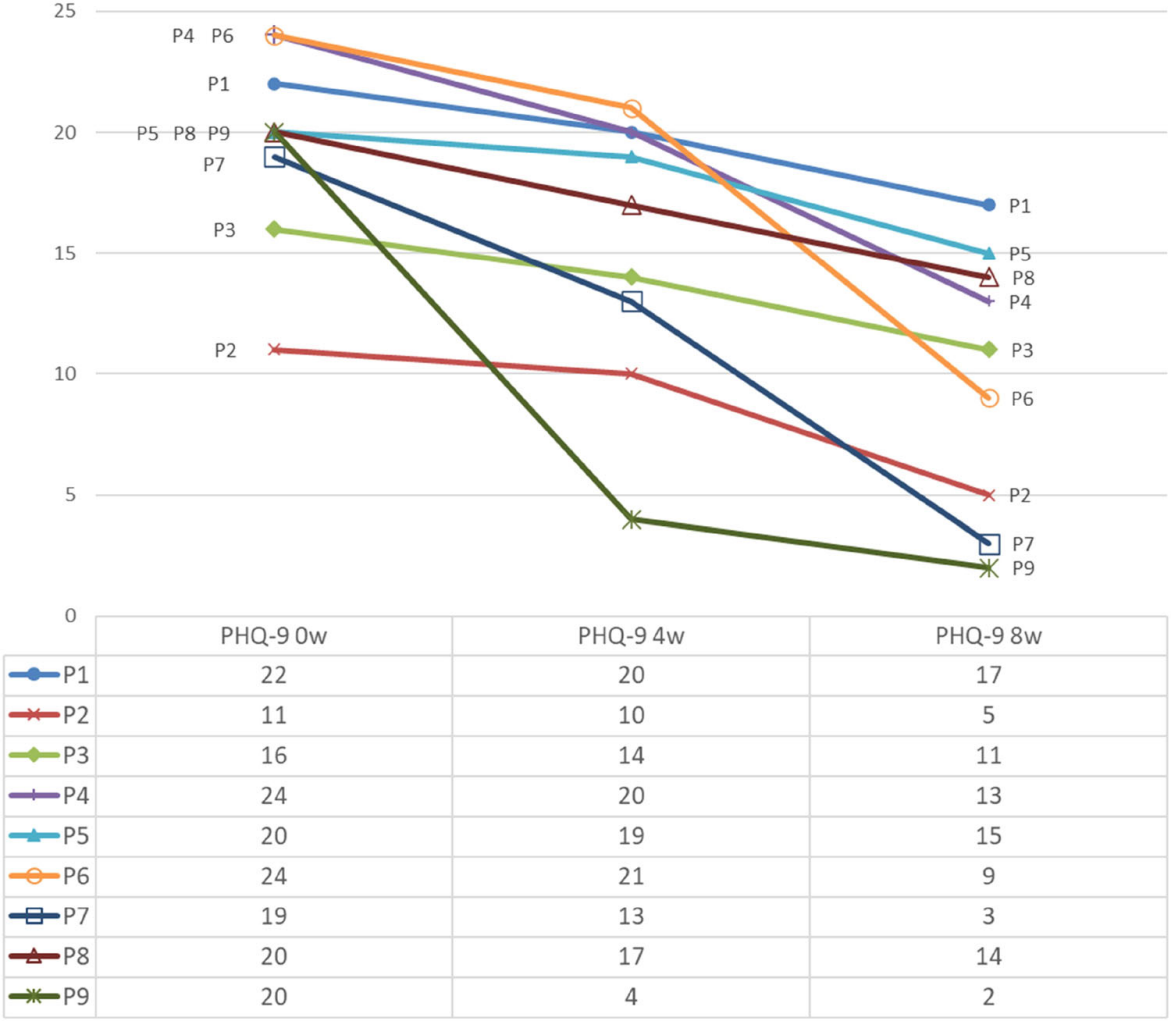
Results by participants.

The results obtained by applying the PHQ‐9 scale at the start of treatment, after four weeks of treatment and at the end of the program (after eight weeks) were compared. All nine participants presented a decrease in their PHQ‐9 score from baseline to four weeks of treatment and further lower after eight weeks of treatment (Figure 1).

At the end of treatment, four participants (44.4%) showed a response to treatment (reduction ≥ 50% of the total PHQ‐9 score in the pre‐treatment assessment) and, of these, two showed remission (PHQ‐9 score < 5). One of these patients who remitted had type II bipolar disorder and was the only one who had not been treated with a tricyclic antidepressant prior to starting treatment.

At baseline, the median PHQ‐9 score indicated a severity level of “severe” depression, which decreased slightly to a “moderately severe” level after four weeks of treatment. Subsequently, it dropped further to a “moderate” level after eight weeks of treatment, with respective medians of 20, 17, and 11 (see Table 2). A statistically significant change was observed between baseline and after eight weeks of treatment (*p* <0.001 for respective pairwise comparisons of Friedman's test, Table 2), also confirmed through the 95% CI for medians not overlapping for baseline and after eight weeks of treatment.

**TABLE 2:** Comparison of PHQ‐9 scores at baseline, week 4 and week 8.

—	Mean (SD) ^1^	Median (IQR) ^2^ 95% CL for median ^3^	*p*‐value ^6 7^
PHQ‐9 0w	19.56 (4.07)	20.00 [19.0; 22.00] [20.00; 24.00]	*p* < 0.001 ^4^ 8w–0w: *p* < 0.001 ^5^ 4w–0w: *p* = 0.102 ^5^ 8w–4w: *p* = 0.102 ^5^
PHQ‐9 4w	15.33 (5.66)	17.00 [13.00; 20.00] [13.00; 20.00]	
PHQ‐9 8w	9.89 (5.46)	11.00 [5.00; 14.00] [5.00; 15.00]	

Abbreviations: 1 SD: Standard Deviation; 2 IQR: interquartile range [P25; P75]; 3 95% CI for median: 95% confidence interval for median; 4 Friedman's Test where *p* = 0.000123409804087; 5 pairwise comparisons tests where 8w–0w: *p* = 0.000066271490996; 6 significance values have been adjusted by the Bonferroni correction for multiple tests.; 7 effect sizes Kendall's W = 1.00, show perfect perfect agreement in the ranking pattern, mainly due to small sample sizes; Effect sizes rank‐biserial correlations for all pairwise comparisons showed very large effects: baseline to 4 weeks (r_rb = 1.00), baseline to eight weeks (r_rb = 1.00), and four weeks to eight weeks (r_rb = 0.97).

At baseline, most patients were at a “severe” depression class level (six out of nine patients), a situation which are inverted after four weeks of treatment (three out of nine patients), culminating with none of the patients being at a “severe” depression class level patients after eight weeks of treatment. The different situation at baseline and eight weeks of treatment was statistically significant (*p* = 0.008 for respective pairwise comparisons of Cochran's Q Test, Table 3).

**TABLE 3:** Comparison of PHQ‐9 and PHQ‐9 item 9 binary recorded classes frequencies at baseline, week 4 and week 8.

—		Freq.	%^a^	*p*‐value ^4^
**PHQ‐9 – severe vs. lower depression severity classes ^5^**				
PHQ‐9 0w	Moderately severe or lower	3	33.33%	*p* = 0.011 ^2^ 8w–0w: *p* = 0.008 ^3^ 4w–0w: *p* = 0.401 ^3^ 8w–4w: *p* = 0.401 ^3^
	Severe	6	66.67%	
PHQ‐9 4w	Moderately severe or lower	6	66.67%	
	Severe	3	33.33%	
PHQ‐9 8w	Moderately severe or lower	9	100.00%	
	Severe	0	0.00%	
PHQ‐9 – item 9 recurrent thoughts of death or suicidal ideation ^6^				
PHQ‐9 0w	No	1	11.11%	*p* = 0.015 ^2^ 8w–0w: *p* = 0.085 ^3^ 4w–0w: *p* = 1.00 ^3^ 8w–4w: *p* = 0.019 ^3^
	Yes	8	88.89%	
PHQ‐9 4w	No	0	0.00%	
	Yes	9	100.00%	
PHQ‐9 8w	No	5	55.56%	
	Yes	4	44.44%	

*Note*: ^a^ Percentages should be interpreted with caution, given the small sample size; ^2^ Cochran's Q Test; ^3^ Pairwise Comparisons Tests; ^4^ Significance values have been adjusted by the Bonferroni correction for multiple tests; ^5^ Confidence intervals (CI) for pairwise differences in proportions (Newcombe method): 8w–0w Δ = 0.67, 95%CI [0.23, 0.88]; 4w–0w Δ = 0.33, 95%CI [–0.05, 0.59]; 8w–4w Δ = 0.33, 95%CI [–0.03, 0.65]; ^6^ CI for pairwise differences in proportions (Newcombe method): 8w–0w Δ = –0.44, 95%CI [–0.69, –0.09]; 4w–0w Δ = 0.11, 95%CI [–0.20, 0.44]; 8w–4w Δ = –0.56, 95%CI [–0.81, –0.14].

Regarding item 9 of the PHQ‐9 instrument, which assesses the presence of recurrent thoughts of death or suicidal ideation, only one patient at baseline reported no such thoughts. After four weeks of treatment, all patients confirmed having such thoughts. However, after eight weeks of treatment, slightly over half the patients reported not having such thoughts (five out of nine patients). The different results after four and eight weeks of treatment were statistically significant (*p* = 0.019 for respective pairwise comparisons of Cochran's Q Test, Table 3).

Regarding depression remission, only two patients presented a PHQ‐9 score below 5 after eight weeks of treatment. This would correspond to a depression remission rate of 22.2% after eight weeks of treatment.

Considering the treatment response rate, only two patients exhibited a reduction of 50% or more in their PHQ‐9 score from four to eight weeks of treatment, but four patients showed a reduction of 50% or more in their PHQ‐9 score from baseline to the end of the eight‐week period. This corresponds to a treatment response rate of 44.4%. In our study, no dropouts were observed.

### 3.3 Post‐Treatment Follow‐Up

The participants were clinically assessed in three follow‐up periods: from zero to three months, from three to six months, and from six to 12 months. For this analysis, it was only possible to include a sample of seven participants since one of the participants was offered ECT after the end of treatment and the other had not yet had a follow‐up. The time to relapse after the end of the program was assessed based on clinical records and follow‐up consultations. The time to relapse after the end of the program was assessed retrospectively through clinical records and follow‐up consultations. As no standardized clinical scales were applied post‐treatment, relapse was determined based on the clinical judgment documented by the assistant psychiatrist. Specifically, participants were considered to have relapsed if their psychiatrist adjusted their antidepressant therapy—by initiating a new medication, switching treatments, or increasing the dosage—due to worsening of depressive symptomatology.

We found that only two of the seven participants had a worsening of mood that required adjustment of antidepressant therapy within three months of the end of treatment. Within the group of five participants who did not relapse in the first three months, three of them had a relapse of symptoms between three and sic months after the end of treatment. Of the remaining two participants, neither had a relapse of depressive symptoms during the first year after treatment.

The follow‐up results showed that most patients (*n* = 5) relapsed within six months of the end of treatment (see supplementary material 1).

## 4 Discussion

Comparing our results with studies that used ketamine without a psychotherapeutic component in patients with depression, we found a response rate higher than that reported by Sakurai et al. (18.5%) and lower than that presented by McInnes et al. (53.6%) (Sakurai et al. 2020, McInnes et al. 2022). Both studies used patient samples from clinical treatment settings. As in our sample, Sakurai et al. included patients who had not responded to multiple antidepressants, and 69% presented at least one comorbid psychiatric disorder, compared with 44.4% in our group. As suggested by Sakurai et al. in their interpretation of low response rates, we also believe that our sample may reflect a high level of treatment resistance, which may translate into lower response rates to treatment in comparison with other studies due to our strict inclusion criteria regarding the number of failed antidepressant attempts, classes of antidepressants used and the use of potentiation strategies (Sakurai et al. 2020); Their study, however, had some limitations: its retrospective design allowed treating psychiatrists to modify concurrent antidepressants or psychotherapeutic regimens as needed, which limits the ability to isolate the effects of ketamine. Additionally, a high dropout rate—possibly linked to treatment costs—may also have influenced outcomes.

In contrast, McInnes et al. reported higher response rates in a large community sample. However, the absence of detailed psychiatric and medication history—such as the number of failed antidepressant trials—limits interpretation, particularly concerning treatment refractoriness. Only 537 of 9016 patients had data available for analysis, raising concerns about selection bias. The use of self‐reported outcomes and lack of randomization further limit internal validity. These methodological differences, alongside distinct sample characteristics and treatment protocols, likely contribute to variability in outcomes across studies. McInnes et al. found that 66.3% of the participants reported suicidal ideation and, among them, 42.7% experienced remission of these thoughts with treatment (McInnes et al. 2022). In our study, at baseline, only one patient denied having suicidal ideation. However, after four weeks of treatment, all patients confirmed having such thoughts. After eight weeks of treatment, 55.56% of the patients reported no suicidal ideation. The temporary increase in suicidal ideation at week 4 underscores the need for close monitoring early in treatment and highlights that clinical improvement may not follow a linear course. While ideation significantly declined by week 8, the early rise raises important risk‐benefit considerations and should be anticipated and addressed as part of clinical management. Consistent with this, a systematic review of 60 ketamine studies reported that suicidality was occasionally observed, sometimes as delayed symptoms, along with one suicide attempt case, and that suicidal ideation contributed to drop out of five participants in one of the studies (Short et al. 2018).

At post‐treatment follow‐up, McInnes et al. reported that 80% of patients maintained a response to treatment at four weeks and 60% at eight weeks (McInnes et al. 2022). In our study, we found that 71% remained stable at 12 weeks after treatment. While we cannot determine whether this longer maintenance period is due to differences in treatment models—namely, ketamine combined with psychological intervention versus pharmacological treatment alone—it is a noteworthy observation. Nevertheless, 71% of patients in our sample experienced relapse within six months. Although this finding must be interpreted cautiously due to the small follow‐up sample size (*n* = 7), it highlights the need to explore strategies to sustain therapeutic benefits over time. Overall, these findings suggest that our response rate is broadly comparable to those reported in ketamine‐only studies (Sakurai et al. 2020). However, without a ketamine‐only or control group, we cannot determine whether the psychotherapeutic component contributed to the observed effects, and it is possible that improvements reflect the pharmacological action of ketamine alone. Importantly, relapses in our study were pragmatically defined as the treating psychiatrist's decision to adjust antidepressant therapy due to worsening of depressive symptomatology. While this reflects real‐world clinical practice, it remains a subjective measure in the absence of standardized post‐treatment scales.

In 2019, Dore et al. conducted a study involving patients receiving ketamine‐assisted therapy for various psychiatric disorders and found that average scores on the beck depression inventory decreased from moderate to mild levels over the course of therapy (Dore et al. 2019). In our study, participants presented with more severe symptoms (PHQ‐9 scores indicating severe depression), improving to a moderate level after eight weeks. Despite our patients initially scoring higher on depression measures compared to those in the Dore et al. study, we observed a statistically significant change from baseline to the end of treatment. The Dore study had important limitations, namely that it included a diagnostically heterogeneous sample. In contrast, our study focused exclusively on patients with treatment‐resistant depression. This shift in PHQ‐9 category, from “severe” to “moderate,” reflects a reduction in symptom burden that is often considered clinically meaningful (Kroenke 2012, Turkoz et al. 2021)

These divergent findings across real‐world studies highlight the inconsistency in outcomes reported in the ketamine literature. This variability is likely influenced by sample characteristics, treatment models, study design, and methodological rigor. Another key factor contributing to this heterogeneity is the variability in ketamine administration protocols across studies. Differences in route (e.g., intravenous, intranasal, intramuscular), dosage, and frequency of administration may significantly affect both pharmacodynamic response and clinical outcomes (Terao et al. 2025, Li and Vlisides 2016). Our standardized IV protocol with psychotherapy differs from approaches in many studies and should be considered when comparing outcomes.

Although our focus has been on real‐world studies, broader literature is relevant. For example, a recent meta‐analysis by Marcantoni et al. (Marcantoni et al. 2020) reported an average remission rate of 28% across a heterogeneous set of studies using ketamine, typically without psychotherapy. Although caution is warranted due to our small sample size, our slightly lower remission rate (22.2%)—observed in a clinical setting using a ketamine combined with psychotherapy protocol—suggests that this approach may hold promise for treatment‐resistant populations (Grabski et al. 2022).

In general, uncontrolled ketamine combined with psychotherapy studies are susceptible to placebo effects, as set and setting factors — including patient expectations, social interactions, and individual beliefs — can shape the effects of drugs and other interventions, significantly influencing outcomes (Hartogsohn 2016). Recent studies suggest that the endogenous opioid system contributes to placebo‐related antidepressant effects and may also be involved in ketamine's mechanism of action. Williams et al. showed that pretreatment with naltrexone blocked ketamine's antidepressant response in patients with treatment‐resistant depression, indicating that opioid signaling may be required for ketamine to exert its effects. This finding raises several possibilities: ketamine's therapeutic benefits might partly reflect expectancy‐driven placebo effects; ketamine may activate opioid‐dependent antidepressant pathways that do not rely on placebo; or ketamine and placebo responses may share a common underlying mechanism involving opioid receptor activity (Costi et al. 2016; Williams et al. 2018; Szigeti and Heifets 2024)

In our retrospective analysis of clinical data, where no control condition was used, we likewise cannot exclude the contribution of non‐specific factors to the observed improvements.

The designation of ketamine‐assisted therapy or ketamine combined with psychotherapy could be a topic of debate, particularly concerning which component—ketamine's effect or the psychological intervention—holds greater significance. If the main role of the psychological intervention is an augmentation effect of the benefits of ketamine, the terminology may be inappropriate. It can also be debated whether it is more correct to use the term ketamine combined with psychotherapy or with psychological intervention. Usually, the use of the psychedelic is accompanied by a specific and/or manualized psychotherapeutic intervention (Seybert et al. 2023).

## 5 Conclusion

In our study, despite the severity of symptoms in a sample that had undergone multiple prior pharmacological interventions, we observed an improvement in depressive symptoms.

### 5.1 Limitations

This study has several limitations. The small sample size limits the generalizability of our findings and severely reduces statistical power; therefore, all results should be interpreted as exploratory signals rather than confirmatory evidence. The size of the sample was influenced by several factors: the constraints imposed by the COVID‐19 pandemic; the complexity of the treatment protocol, given the off‐label use of ketamine; and the nature of our treatment model, which involves ketamine‐assisted therapy including psychological intervention, and therefore requires a greater number of resources compared to pharmacological‐only approaches. Second, the absence of a control group—such as a therapy‐only or placebo condition—prevents us from isolating the specific contribution of ketamine from that of the psychotherapeutic component. This limits our ability to determine whether the combined treatment had a greater impact than therapy alone. No formal psychotherapy fidelity assessments were conducted, which limits our ability to evaluate consistency across therapists and to confirm adherence to the ACT/MIND‐based protocol. Our outcome measures were limited to the PHQ‐9 and non‐standardized clinical interviews, as we did not include validated anxiety, functioning, or quality‐of‐life scales, nor clinician‐rated measures such as the HAM‐D or MADRS. Interviews were not conducted with structured diagnostic tools (e.g., SCID), which reduces standardization. Finally, it would be important to have determined what were the most impactful components acting as mediators in the treatment process, such as changes in bodily or narrative self‐experience during ketamine administration (Marguilho et al. 2023).

### 5.2 Further Research

Marguilho et al. proposed that the rapid and transient antidepressant effects of ketamine are mainly due to its acute ability to modulate the reward circuits and sub‐acute ability to increase neuroplasticity, while the dissociative and psychedelic properties are due to dose‐ and context‐dependent disruption of large‐scale functional networks, such as the transient desegregation and disintegration of the salience network and default‐mode network. According to this hypothesis, the use of ketamine integrated into the psychedelic‐assisted psychotherapy model should be associated with better and longer‐lasting therapeutic outcomes (Marguilho et al. 2023). More comparative studies are needed for the treatment of resistant depression between the use of ketamine infusions alone and ketamine‐assisted therapy, including a randomized controlled trial, as well as the inclusion of placebo or expectancy‐control arms and formal psychotherapy fidelity assessment.

Currently, evidence is limited regarding the role of biomarkers in the response to ketamine treatment. Medeiros et al. published a systematic review, which reported that patients who responded to ketamine treatment had a statistically significant increase in brain‐derived neurotrophic factor when compared with pre‐treatment levels, unlike patients who did not respond to treatment. More studies are needed on biological markers that may allow us to identify subgroups of patients who could benefit more from treatment with ketamine (Medeiros et al. 2022).

In 2023, Reif et al. published a study comparing the effect of esketamine with quetiapine when combined with an SSRI or SNRI in resistant depression, showing better results with esketamine (Reif et al. 2023). More recent findings from 2024–2025 provide a nuanced update on esketamine's role. A systematic review concluded that its antidepressant effects appear modest—similar to augmentation with atypical antipsychotics—and raised concerns regarding safety and unclear long‐term risks (Fountoulakis et al. 2025). In contrast, a large randomized clinical trial showed that esketamine nasal spray used as monotherapy produced rapid and clinically meaningful reductions in depressive symptoms compared with placebo, suggesting potential value for patients who do not tolerate or respond to oral antidepressants (Janik et al. 2025). Esketamine is already approved and reimbursed in several countries, and further research comparing the efficacy and cost‐effectiveness of ketamine versus esketamine remains important.

Ketamine‐Assisted Therapy is a safe procedure with significant potential in the treatment of various psychiatric disorders (Figueiredo et al. 2023). It would be important to continue investigating the potential of psychedelic drugs within the psychiatric field. Future studies should further explore the implementation and outcomes of ketamine combined with psychotherapy in public healthcare settings, where resources and infrastructure differ from those in private or research‐based environments.

## Author Contributions

F.A.S. collected the data and wrote some chapters of the article. R.A. and B.P. wrote some chapters of the article. L.N. analyzed the data. F.S. collected the data and was involved in the first draft of the manuscript. J.R. conceptualized the therapy protocol and was involved in the first draft of the manuscript. M.J.H. supervised and coordinated the different stages of the paper writing, and revised the multiple drafts. All authors revised and approved the final draft of the manuscript.

## Funding

The authors have nothing to report.

## Ethics Statement

The reported clinical cases were authorized by the Ethics Committee for Health of the ULS Loures‐Odivelas, Portugal. All the participants signed a written informed consent prior to undergoing treatment. The requirements of the General Data Protection Regulation were followed. This study was conducted under the Helsinki Declaration Code of Ethics.

## Consent

Verbal informed consent was obtained from patients for the purpose of publication.

## Conflicts of Interest

The authors declare no conflicts of interest

## Supporting information




**Supplementary Material**: brb371164‐sup‐0001‐SuppMat.docx

**Supplementary Material**: brb371164‐sup‐0002‐SuppMat.docx

## Data Availability

The data that support the findings of this study are available from the corresponding author upon reasonable request.
