# Supplementary material for: Ketamine Combined With Psychotherapy as a Treatment for Resistant Depression in a Public European Hospital
Source: Brain Behav. 2026 Jan 13;16(1):e71164. doi: 10.1002/brb3.71164 (PMC12796847; doi:10.1002/brb3.71164)
Supplement: Supplementary file 1 — Supplementary Material: brb371164‐sup‐0001‐SuppMat.docx [file BRB3-16-e71164-s001.docx]

SUPPLEMENTARY MATERIAL (SM1)


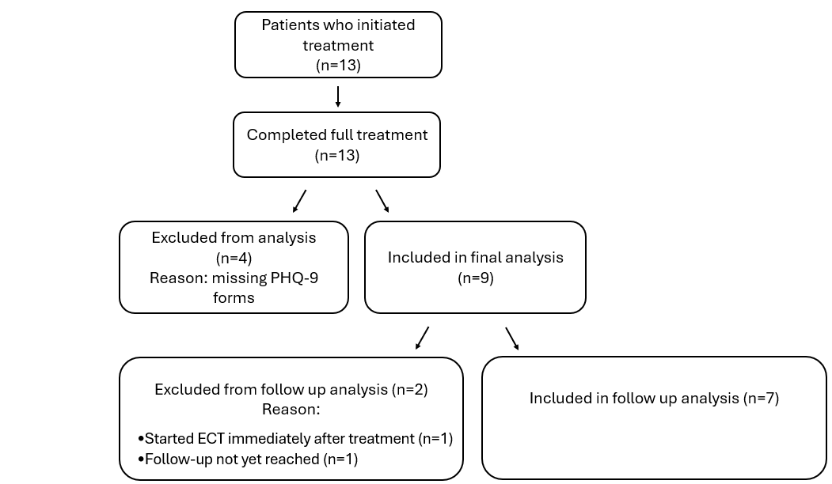


Figure 1 - Patient flow diagram


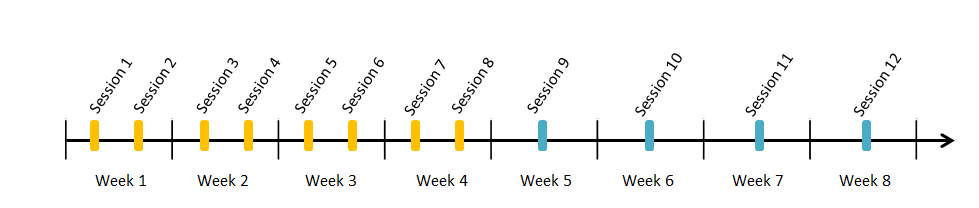


Figure 2 - Ketamine infusion sessions timeline


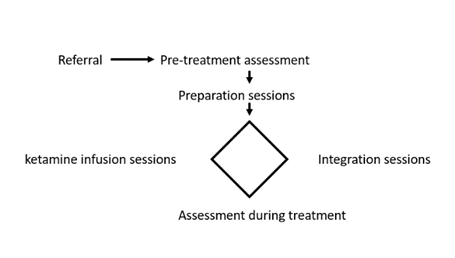


Figure 2 - Treatment flowchart

Table 1 - Ketamine infusion sessions – dosing details by participant

| Patient | Initial dose | Median dose (mg) | Maximum dose (mg) |
| --- | --- | --- | --- |
| 1 | 28.9 mg | 55.0 mg | 66.5 mg |
| 2 | 21 mg | 37.4 mg | 41 mg |
| 3 | 18 mg | 38.8 mg | 50 mg |
| 4 | 16 mg | 23.9 mg | 47 mg |
| 5 | 16 mg | 32.7 mg | 47 mg |
| 6 | 24 mg | 26.3 mg | 38 mg |
| 7 | 17 mg | 42.7 mg | 49.5 mg |
| 8 | 23,45 mg | 40.5 mg | 50.25 mg |
| 9 | 17,3 mg | 38.2 mg | 41.4 mg |

Table 2 -Self-reported adverse events during ketamine treatment (n=9). Values represent the number (%) of patients who experienced each effect at least once, based on repeated session-by-session questionnaires.

| Adverse event | n (%) |
| --- | --- |
| Chest pain | 3 (33.3%) |
| Agitation | 4 (44.4%) |
| Irritability | 3 (33.3%) |
| Anxiety | 4 (44.4%) |
| Negative or despairing thoughts | 3 (33.3%) |
| Somnolence | 6 (66.7%) |
| Dizziness | 5 (55.6%) |
| Visual disturbances | 6 (66.7%) |
| Auditory disturbances | 4 (44.4%) |
| Nausea and vomiting | 2 (22.2%) |
| Increased urinary frequency | 3 (33.3%) |
| Shortness of breath | 3 (33.3%) |
| Hypersalivation | 2 (22.2%) |
| Muscle stiffness | 2 (22.2%) |

Table 3 - clinical information at follow up

| Clinical Information at Follow-up | | | |  |
| --- | --- | --- | --- | --- |
| Follow up - relapse 0-3M | Yes | 2 | 28,57% | |
|  | No | 5 | 71,43% | |
| Follow up - relapse 3-6M | Yes | 3 | 60,00% | |
|  | No | 2 | 40,00% | |
| Follow up - relapse 6-12M | Yes | 0 | 0,00% | |
|  | No | 2 | 100,00% | |

Table 4 - Individual Patient Trajectories: PHQ-9 Total Scores and Item 9 Across Treatment

| PATIENT | PHQ-9 (0w) | PHQ-9 (4w) | PHQ-9  (8w) | PHQ-9 – item 9^1^  (0w) | PHQ-9 – item 9 ^1^ (4w) | PHQ-9 – item 9 ^1^ (8w) |
| --- | --- | --- | --- | --- | --- | --- |
| 1 | 22 | 20 | 17 | 2 | 2 | 2 |
| 2 | 11 | 10 | 5 | 1 | 1 | 0 |
| 3 | 16 | 14 | 11 | 3 | 3 | 3 |
| 4 | 24 | 20 | 13 | 2 | 2 | 1 |
| 5 | 20 | 19 | 15 | 2 | 2 | 1 |
| 6 | 24 | 21 | 9 | 0 | 1 | 0 |
| 7 | 19 | 13 | 3 | 2 | 1 | 0 |
| 8 | 20 | 17 | 14 | 3 | 1 | 0 |

^1^PHQ-9 Item 9 assesses suicidal ideation (“Thoughts that you would be better off dead or of hurting yourself in some way”), scored from 0 to 3.

Table 5 - Primary Diagnosis, Comorbidities and Prior Antidepressant Classes

| PATIENT | Primary diagnosis  (ICD-10) | concomitant psychiatric disorder  (ICD-10) | Classes of Antidepressants Previously Tried | Classes of Augmentation Strategies Previously Used |
| --- | --- | --- | --- | --- |
| 1 | F 33.2 Recurrent depressive disorder, current episode severe without psychotic symptoms | F60.7 Dependent personality disorder | SNRI; NDRI; TCA | Lithium; SGA |
| 2 | F32.2 Severe depressive episode without psychotic symptoms | NA | SNRI; TCA | VPA; SGA |
| 3 | F33.1 Recurrent depressive disorder, current episode moderate | F60.3 Emotionally unstable personality disorder | NaSSA; SNRI; TCA | VPA; LMT; SGA |
| 4 | F33.1 Recurrent depressive disorder, current episode moderate | F43.1 Post-traumatic disorder | SSRI; SARI; TCA | AP; SGA |
| 5 | F33.2- Perturbação depressiva major, recorrente, grave sem características psicóticas; | NA | SSRI; SNRI; TCA | NaSSA; Lithium; SGA |
| 6 | F33.1 Recurrent depressive disorder, current episode moderate | NA | SSRI; SNRI; TCA | Lithium; SGA |
| 7 | F31.81 Bipolar II disorder | F10.1 Mental and behavioral disorders due to use: harmful use | NDRI; SSRI; SNRI; | Lithium; SGA |
| 8 | F33.2 Recurrent depressive disorder, current episode severe without psychotic symptoms | NA | SSRI; SNRI; TCA | SGA |

**Abbreviations:** ICD= International Classification of Diseases. NA = Not Applicable. SSRI = Selective Serotonin Reuptake Inhibitor; SNRI = Serotonin–Norepinephrine Reuptake Inhibitor; TCA = Tricyclic Antidepressant; NDRI = Norepinephrine–Dopamine Reuptake Inhibitor; NaSSA = Noradrenergic and Specific Serotonergic Antidepressant; SARI = Serotonin Antagonist and Reuptake Inhibitor. VPA = valproate; LMT = lamotrigine; SGA = second-generation antipsychotic.
