# Supplementary material for: Ketamine Combined With Psychotherapy as a Treatment for Resistant Depression in a Public European Hospital
Source: Brain Behav. 2026 Jan 13;16(1):e71164. doi: 10.1002/brb3.71164 (PMC12796847; doi:10.1002/brb3.71164)
Supplement: Supplementary file 2 — Supplementary Material: brb371164‐sup‐0002‐SuppMat.docx [file BRB3-16-e71164-s002.docx]

SUPPLEMENTARY MATERIAL – PSYCHOTHERAPEUTIC PROTOCOL DETAILS (SM2)

1. **Preparation Session**

Total duration: 2 hours

General Objectives of Preparation:

- To promote a mindset that facilitates psychedelic experiences with therapeutic value, namely:
- Establish a therapeutic alliance and build trust.
- Identify mechanisms of psychological inflexibility.
- Assess and align expectations regarding the experience.
- Facilitate surrender to the experience.
- Establish intentions for the therapy.

Session structure:

1. Depression narrative – 30 min
2. Psychoeducation about the experience – 40 min
3. Facilitating surrender – 30 min
4. Establishing intentions – 20 min
5. **Depression Narrative** (30 min)

- Obtain a personal narrative of depression and prior treatments.
- Identify mechanisms of psychological inflexibility:
- Cognitive fusion: rigid beliefs (“I am useless”, “I can’t do anything”).
- Experiential avoidance: emotional suppression, isolation, avoidance of people and situations.
- Past dominance: rumination, excessive search for reasons behind suffering.
- Conceptualized self: self-criticism, unrealistic expectations, restricted roles.
- Identify personal values.
- Assess attitude toward improvement: active willingness vs. passive desire → promote committed action.

1. **Psychoeducation About the Experience (40 min)**

- Explore prior experiences and expectations about psychedelics.
- Explain that during the ketamine session, one may have unusual experiences, that these may hold meaning, that meaning may not be immediately apparent, and that there is no need to be afraid.
- Provide examples of possible common experiences
- Offer music during the session and explain its therapeutic role.
- Prepare for challenging experiences
- Explain that after the first sessions, emotions long suppressed may resurface.
- Emphasize the importance of integration and concrete action after the experience;

1. **Facilitating Surrender** (30 min)

- Reinforce the importance of curiosity, acceptance, trust in the process, and openness to whatever arises.
- Assure that the patient will never be alone and can request help from the team at any time.
- Use the “Chinese finger trap” exercise as a metaphor for surrender as liberation.
- Select and train a grounding exercise to calm during the experience.

1. **Establishing Intentions** (20 min)

- What are my most important challenges right now?
- What would I like to feel during the experience?
- The therapy will be successful when…?

**Practical Recommendations for the Patient**

- Avoid alcohol for 48 h before and after each ketamine session.
- No food intake for 4 h before treatment.
- Wear comfortable clothing.
- Ensure a companion for leaving the hospital after the session.
- Bring a journaling notebook to record experiences and therapeutic process between sessions.

**B. Psychological Support During the Experience**

Total duration: ~3 hours

1. Reception: 30 min
2. Support during the experience: 1.5 h
3. Recovery and discharge: 1 h

1. **Reception** (30 min)

- Welcome the patient and introduce the team. Ask about any medical events or changes since the last treatment. Ensure 4-hour fasting and confirm the presence of an escort for discharge. Explain or remind the patient of the procedures.
- Administer the PHQ-9 (Patient Health Questionnaire-9).
- Assure the patient that they will be supported at all times. Review key preparation instructions:
- Remind the patient of their intentions, while allowing flexibility and non-attachment to them.

2. **Non-Intrusive Support During the Experience** (1.5 h)

- The patient leads the experience. The guide remains vigilant, in the background, ready to support, reassure, and facilitate surrender if needed.
- Check-in periodically: Reaffirm presence and readiness to help if needed.
- Responding to requests to talk: Listen empathetically, then gently redirect the patient to introspection. Leave deeper processing for integration sessions. Remind the patient (when appropriate) of the importance of surrender, curiosity, acceptance, and openness to whatever arises.
- Responding to intense emotional states:Distinguish between panic and resistance.
- Use music.

3. **Recovery and Discharge** (1 h)

- Allow the patient to briefly share about the experience, validating their account, but defer deeper processing to integration sessions.
- Offer food.
- Confirm clinical stability and absence of adverse effects.
- Encourage the patient to use their journaling notebook at home or the next day to write or draw about the experience.
- Set the date for the next ketamine session and integration session.
- Ensure the patient leaves accompanied.

**C. Integration Sessions**

Total duration: 1h-1h30/week

**Main objective:** Transform the learning from the psychedelic experience into concrete, committed changes in the patient’s life, improving functionality both internally and externally in daily living.

The integration process is organized into two complementary phases. The first phase focuses on recalling, expressing, and allowing the psychedelic experience to unfold (Center, Remember, Express, Let Arrive). In the second phase, the material that emerges—including symbolic or mystical-type insights—is explored to clarify its personal meaning. After identifying a significant moment or insight, the patient is supported in understanding what it represents for their life and in identifying the value it reflects. Once this value is clear, it is translated into a concrete and realistic action to be implemented in daily life, establishing a coherent progression from experience to insight, from insight to value, and from value to action.

STRUCTURE – “INTEGRATION CURVE”

Phases:

1. **Center** – Bring focus to the present moment and recall the intention.
2. **Remember** – Evoke and revisit the experience or part of it.
3. **Express** – Convey the remembered experience through artistic media or words.
4. **Let Arrive** – Allow insights and future visions to emerge from the experience.
5. **Connect** – Link insights to life history, intention, psychological flexibility, and values.
6. **Act** – Promote concrete and committed actions aligned with flexibility and values.
7. **Centering**

- Abdominal breathing.
- Attention to bodily sensations.
- Other mindfulness techniques.
- Music.

1. **Remember**

- Guided meditation.
- Rereading the journaling notebook.
- Free-association writing.
- Musical journey.
- Surrender exercises.

1. **Express**

Express the remembered experience through:

- Collage
- Watercolor painting
- Drawing
- Drama / body statues
- Music / dance
- Writing
- Speaking

1. **Let Arrive**

- Allow the arrival of insights based on the assumption that, in the psychedelic state, the patient accesses intrinsic wisdom.
- Emphasize future visions rather than elaborations about the past, facilitating the path toward concrete and committed action.
- Use specific exercises to receive new insights and future visions:

- “Life Road” exercise.

- “Tombstone” exercise.

- List of values.

1. **Connect – Act**

The primary objective of integration is to translate the learning acquired from the psychedelic experience into concrete and committed changes in the patient’s life, improving functionality both internally and externally in daily living. In this integration phase, the aim is to connect the experience and the insights that arose from it with:

- Life history**:** Therapists facilitate reappraisal of the past and detachment from it.
- The original therapy intention**:** Therapists ask how this learning can help fulfill that intention.
- Psychological flexibility mechanisms, particularly those most affected by their inflexible counterparts in the ACT model. Therapists highlight and reinforce instances of psychological flexibility experienced.
- New, rediscovered, or reprioritized values and visions for the future. Therapists emphasize the emergence of new values and the importance of taking action aligned with them.
- The focus of this intervention should remain on the experience and the learning that resulted from it. This is a brief therapy designed to optimize the therapeutic value of the psychedelic experience.
- In this stage, therapists promote concrete and committed action aligned with values, future visions, psychological flexibility mechanisms, insights, other learnings, and reappraisal or detachment from the past.
- Goals should be small, concrete, and realistic, enabling the person to commit to them. In this way, they can actually apply them, experience gratification, empowerment, and observe change.
- The therapist explains that it is not necessary to change one’s entire life; actions are prioritized and undertaken one at a time.

No formal patient comprehension metrics were employed during the integration phase.

**D. Therapist Qualifications and Training**

All therapists involved in the application of the KAP protocol were board-certified mental health professionals in various capacities. The main therapists received specific training in the protocol, which comprised six hours of theoretical instruction on KAP and the manualized protocol, along with practical training through observation and supervised practice.
